# Prognostic significance of the pretreatment pan-immune-inflammation value in colorectal cancer patients: an updated meta-analysis

**DOI:** 10.3389/fonc.2025.1599075

**Published:** 2025-07-24

**Authors:** Jing Li, Huayang Pang, Hao Sun, Xiaoyu Liu

**Affiliations:** Department of Gastrointestinal Cancer Center, Chongqing University Cancer Hospital, Chongqing, China

**Keywords:** colorectal cancer, pan-immune-inflammation value, overall survival, disease-free survival, cancer-specific survival, meta-analysis

## Abstract

**Background:**

The prognostic importance of the pretreatment pan-immune-inflammation value (PIV) in colorectal cancer has been extensively documented, yet its role remains unclear. This study aims to conduct an updated meta-analysis to elucidate the relationship between the pretreatment PIV and long-term survival outcomes among patients diagnosed with colorectal cancer.

**Methods:**

A systematic literature review was performed in PubMed, Embase, Web of Science and CNKI to identify eligible studies from inception to January 18, 2025. The primary endpoints evaluated were survival outcomes. Hazard ratios (HRs) along with their corresponding 95% confidence intervals (CIs) for survival outcomes were extracted. A random-effects model was utilized to synthesize the findings. All statistical analyses were conducted using R software, version 4.2.1.

**Results:**

Out of 81 identified studies, a total of 14 retrospective studies including 6,192 colorectal cancer patients were ultimately included. In this meta-analysis, the pooled results demonstrated that patients with higher PIV exhibited significantly poorer overall survival (11 studies, HR=1.95; 95%CI:1.64-2.31; P<0.01; I^2^ = 34%) and disease-free survival (10 studies, HR= 1.89; 95% CI: 1.48-2.41; P < 0.01; I^2^ = 66%). Furthermore, evidence pooled from two studies demonstrated that PIV may be an independent prognostic factor for cancer-specific survival (HR= 2.61; 95% CI: 1.56-4.38; P < 0.01; I^2^ = 0%).

**Conclusion:**

Our study reveals that the pretreatment PIV can serve as a valuable biomarker for predicting long-term survival outcomes in patients with colorectal cancer, which may have important clinical implications for personalized treatment strategies.

## 1 Background

Colorectal cancer (CRC) continues to rank as the third most commonly diagnosed malignancy and the second leading cause of global cancer-related mortality (1). Despite remarkable progress in surgical techniques, chemotherapeutic regimens, radiotherapy protocols, targeted therapies, and immunotherapeutic interventions for CRC patients, clinical outcomes remain suboptimal (2). To date, the Tumor-Node-Metastasis (TNM) classification system has been universally acknowledged as the cornerstone for stratifying prognostic risks in CRC. However, extensive evidence demonstrates considerable heterogeneity in patient outcomes even within the same TNM stage, particularly in stages II and III (3). This variability underscores the limitations of relying solely on TNM staging to fully predict the prognostic outcomes. As such, there is an imperative need to identify robust biomarkers capable of refining risk stratification and pinpointing individuals at high risk of adverse prognoses.

A large amount of evidence underscores the pivotal role of host inflammation and immune status in modulating the progression, treatment responsiveness, and survival trajectories of cancer patients (4, 5). Drawing upon this insight, a number of inflammation/immune-related biomarkers has emerged to forecast clinical outcomes in oncology, including the monocyte-to-lymphocyte ratio (MLR) (6), neutrophil-to-lymphocyte ratio (NLR) (7), and platelet-to-lymphocyte ratio (PLR) (8). Recently, a novel prognostic biomarker—the pan-immune-inflammation value (PIV)—has captured the attention of clinicians worldwide (9, 10). By integrating neutrophils, platelets, monocytes, and lymphocytes into a single metric, PIV has demonstrated superior prognostic accuracy compared to its simpler counterparts, such as NLR, NLR, and PLR (9). Specifically, PIV is calculated via the formula: serum neutrophil × platelet × monocyte ÷ lymphocyte, a methodology first introduced by Fuca et al. (11) in 2020 as a prognostic index for metastatic colorectal cancer patients undergoing chemotherapy combined with targeted therapy. Subsequently, the prognostic utility of PIV has been progressively investigated across various clinical settings of CRC patients (12–14). In 2022, Yang et al. (15) conducted the first meta-analysis encompassing six studies, preliminarily demonstrating the prognostic significance of PIV in CRC patients. Nevertheless, they conceded that the limited number of included studies rendered the prognostic implications of PIV in CRC somewhat ambiguous. In light of the burgeoning recent literature, we undertook an updated meta-analysis to further illuminate the correlation between pretreatment PIV and long-term oncological outcomes in CRC patients.

## 2 Methods

### 2.1 Search strategy

The present meta-analysis was conducted in accordance with the Preferred Reporting Items for Systematic Reviews and Meta-Analyses (PRISMA) guidelines (16). A systematic and comprehensive search for relevant studies was performed across multiple databases, including PubMed, Embase, CNKI, and Web of Science, spanning from their inception to January 18, 2025. The search strategy involved a combination of predefined keywords: (pan-immune-inflammation value) AND (((colorectal) OR (colon) OR (rectum) OR (rectal)) AND ((cancer) OR (tumor) OR (carcinoma))). No restrictions were imposed on language during the search process. Additionally, the reference lists of the included studies were thoroughly examined to identify further relevant reports. Two investigators (L-J and P-HY) independently executed the search procedure.

### 2.2 Study selection

The inclusion criteria were as follows: (1) Studies investigating the association between the pretreatment PIV and survival outcomes in patients with CRC, including overall survival (OS), recurrence-free survival (RFS), disease-free survival (DFS), progression-free survival (PFS) and cancer-specific survival (CSS); (2) Hazard ratios (HRs) along with their 95% confidence intervals (CIs) were either directly reported or could be calculated based on the original survival curves; (3) The specific cut-off value of the PIV was clearly defined. The exclusion criteria were as follows: (1) Studies that failed to provide distinct data for CRC patients; (2) Case reports, reviews, conference abstracts, and correspondence; (3) Overlapping datasets.

### 2.3 Data extraction and quality assessment

Two independent reviewers (L-J and P-HY) performed data extraction and conducted cross-verification of all results. The extracted data encompassed essential information, including the first author’s name, publication year, study period, country, study design, blood sampling time, whether diseases affecting biomarker testing were excluded, sample size, cut-off value determination method, cut-off value of the PIV, and clinicopathological characteristics such as age, sex, primary treatment, tumor stage, tumor location, survival outcomes, and follow-up duration. The quality of the included studies was rigorously evaluated using the Newcastle-Ottawa Scale (NOS) (17), which consists of eight predefined items. Each study was assigned a final score ranging from 0 to 9 based on a comprehensive assessment; scores of 7–9 were considered indicative of high-quality research.

### 2.4 Statistical analysis

In this study, since RFS, PFS, and DFS share similar endpoints, they were collectively analyzed as a single outcome measure (DFS), consistent with previous literature (18, 19). The HRs along with their corresponding 95% CIs were used as the effect size for these survival outcomes. Statistical heterogeneity among the included studies was assessed using the I²statistic, and I²≥ 50% was considered indicative of significant heterogeneity. A random-effects model was employed to synthesize HRs during the meta-analysis due to the substantial clinical heterogeneity across studies. Subgroup analysis was conducted to evaluate the robustness of the pooled results. Additionally, sensitivity analysis was performed to explore the source of heterogeneity in the pooled results when significant heterogeneity was present. Begg’s funnel plot was utilized to assess potential publication bias. For pooled outcomes exhibiting significant publication bias, the trim-and-fill method was further applied. A two-tailed P value less than 0.05 was deemed statistically significant. All statistical analyses were conducted using R software, version 4.2.1.

## 3 Results

### 3.1 Study characteristics

**Figure 1:**
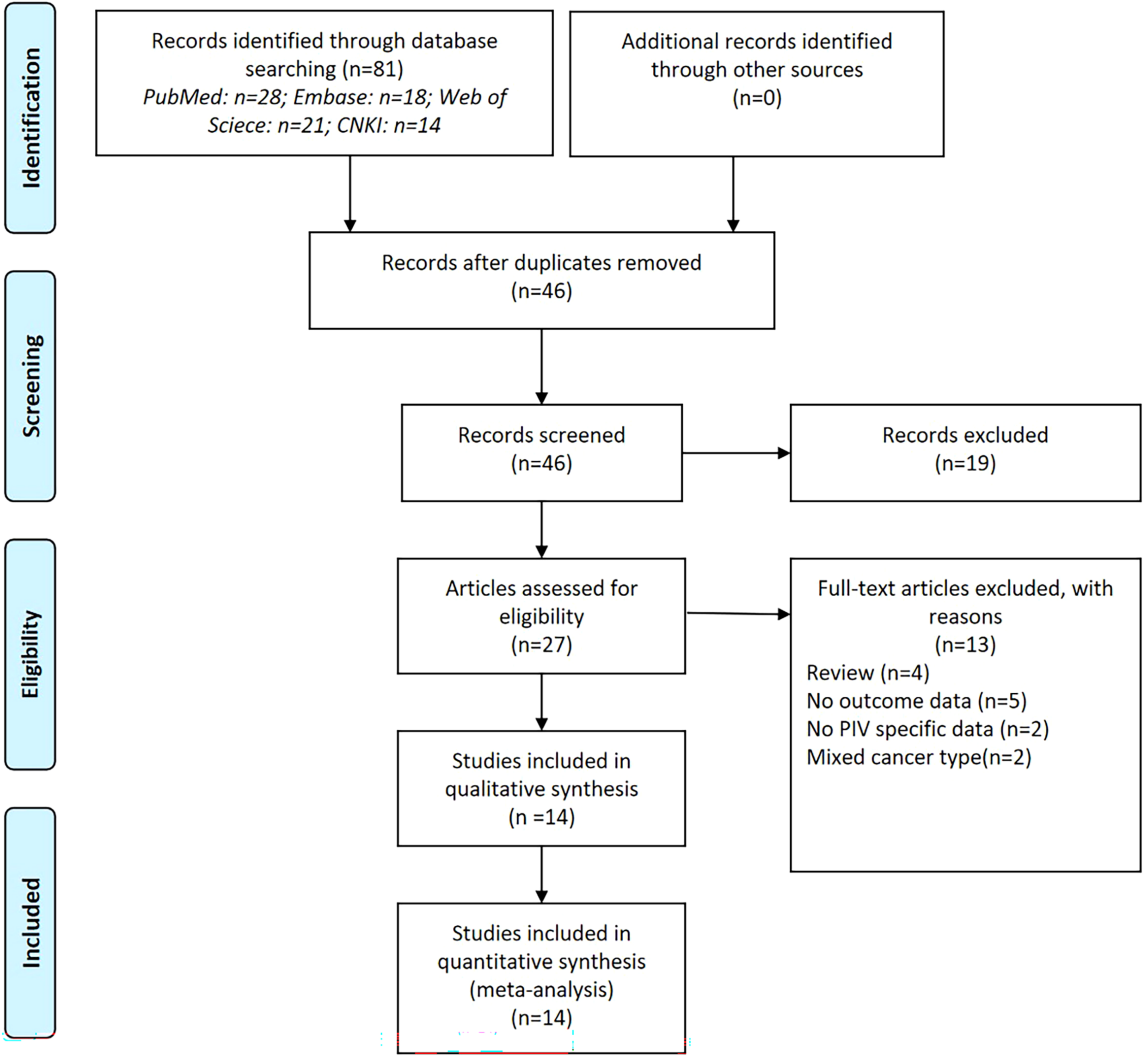
The PRISMA Flowchart of study selection.

The database search resulted in a total of 81 records, as illustrated in **Figure 1**. Following a rigorous assessment of titles, abstracts, and full texts, 14 studies (11–14, 20–30) were ultimately selected for inclusion in this analysis. **Tables 1** and **2** presented detailed summaries of the basic characteristics and survival outcomes of these studies, respectively. In summary, this meta-analysis included a total of 6,192 patients from six countries: China, Japan, Italy, Korea, Turkey, and Spain. The publication years spanned from 2020 to 2024, with sample sizes ranging from 86 to 801 participants. Among the included studies, 10 studies focused on colorectal cancer, 2 studies on colon cancer, and 2 studies on rectal cancer. Concerning primary treatment modalities, 10 studies involved surgical interventions, 3 studies involved systemic treatments, and 1 study focused on neoadjuvant therapy. Regarding survival endpoints, 11 studies evaluated OS, 5 studies assessed DFS, 3 studies examined PFS, 2 studies analyzed RFS, and 2 studies evaluated CSS. Notably, all studies exhibited high quality, with NOS scores ranging from 6 to 7 (as shown in **Table 1**, **Supplementary Table S1**).

**Table 1:** Basic information of included cohorts.

References	Country	Study design	Study interval	Tumor location	Sample size, n (High PIV group: Low PIV group)	Blood sampling time	Excluding patients with diseases affecting biomarker test	NOS
Fuca, 2020 (11)	Italy	M; R	2008-2018	Colorectal cancer	438 (230:208)	Before treatment	NR	6
Corti, 2021 (12)	Italy	M; R	2014-2020	Colorectal cancer	163 (63:100)	Within 1 week before treatment	NR	6
Perez-Martelo, 2022 (13)	Spain	S; R	2015-2018	Colorectal cancer	130 (70:60)	Within 1 month before treatment	NR	7
Sato R, 2022 (26)	Japan	S; R	2013-2020	Colorectal cancer	86 (63:23)	Before treatment	Yes	6
Sato S, 2022 (14)	Japan	S; R	2000-2019	Colorectal cancer	758 (190:568)	Before treatment	Yes	7
Efile, 2023 (20)	Turkey	S; R	2008-2016	Colon cancer	304 (152:152)	Within 2 weeks before treatment	NR	7
Liang, 2023 (23)	China	S; R	2013-2016	Colorectal cancer	753 (374:379)	Within 1 week before treatment	NR	7
Feng, 2024 (21)	China	S; R	2016-2021	Colorectal cancer	108 (52:56)	Within 1 week before treatment	Yes	7
Liu, 2024 (24)	China	S; R	2018-2019	Colorectal cancer	172 (65:107)	Before treatment	Yes	7
Ni, 2024 (25)	China	S; R	2012-2020	Colorectal cancer	437 (206:231)	Within 1 week before treatment	Yes	7
Seo, 2024 (27)	Korea	S; R	2016-2020	Colorectal cancer	203 (118:85)	Within 2 days before treatment	NR	7
Shen, 2024 (28)	China	M; R	2015-2020	Rectal cancer	215 (62:153)	Within 2 weeks before treatment	Yes	7
Wang (1), 2024 (30)	China	M; R	2014-2021	Left-sided Colon cancer	801 (282:519)	Before treatment	NR	7
Wang (2), 2024 (30)	China	M; R	2014-2021	Right-sided Colon cancer	709 (482:227)	Before treatment	NR	7
Wang (3), 2024 (29)	China	S; R	2018-2021	Rectal cancer	679 (101:578)	Within 1 week before treatment	NR	7
Wang (4), 2024 (29)	China	S; R	2015-2017	Rectal cancer	236 (32:204)	Within 1 week before treatment	NR	7

M, multiple centers; S, single center; R, retrospective study; PIV, pan-immune-inflammation value; NOS, Newcastle-Ottawa Scale; NR, not reported.

**Table 2:** Clinical and survival information of included cohorts.

References	Age, years (median or mean)	Sex (male/female)	Selection method for cut-off value	Cut-off value	Tumor stage	Primary treatment	Survival outcomes	Multivariate analysis	Median follow-up time, months
Fuca, 2020 (11)	62 (IQR, 53-68)	275/163	MSR	380	IV	Chemotherapy and target therapy	OS; PFS	Yes; Yes	38.4 (IQR, 27.4-50.9)
Corti, 2021 (12)	NR	90/73	MSR	492	IV	Immunotherapy	OS; PFS	Yes; Yes	31
Perez-Martelo, 2022 (13)	68.8 (Range, 26-88)	96/34	MSR	380	IV	Chemotherapy	OS; PFS	Yes; Yes	NR
Sato R, 2022 (26)	70 (Range, 37-93)	50/36	ROC	209	I-III	SEMS and surgery	CSS; RFS	Yes; Yes	35 (Range, 1-104)
Sato S, 2022 (14)	NR	466/292	ROC	376	I-III	Surgery	OS; RFS	Yes; Yes	63.5
Efile, 2023 (20)	62 (Range, 19-91)	182/122	Median	491	II-III	Surgery	OS; DFS	Yes; Yes	NR
Liang, 2023 (23)	NR	473/280	ROC	231	I-IV	Surgery	OS	Yes	NR
Feng, 2024 (21)	61.40 ± 11.10	63/45	ROC	464.9	I-IV	Surgery	OS	Yes	NR
Liu, 2024 (24)	NR	108/64	X-tile	265.75	I-IV	Surgery	OS	Yes	NR
Ni, 2024 (25)	NR	255/182	ROC	463.7	I-IV	Surgery	OS	Yes	NR
Seo, 2024 (27)	65.89	139/64	Contal and O’Quigley methods	155.9	I-IV	Surgery	OS; DFS	Yes; Yes	40.8
Shen, 2024 (28)	58(Range, 25-79)	132/83	X-tile	454.7	II-III	Neoadjuvant chemoradiotherapy and surgery	OS; DFS	Yes; Yes	NR
Wang (1), 2024 (30)	63(IQR, 54-70)	306/495	MSR	227.84	I-III	Surgery	DFS	Yes	44.17 (IQR, 29.67-62.32)
Wang (2), 2024 (30)	64 (IQR, 55-72)	332/377	MSR	145.99	I-III	Surgery	DFS	No	44.17 (IQR, 29.67-62.32)
Wang (3), 2024 (29)	64 (IQR, 56-70)	413/266	MSR	363.906	I-III	Surgery	CSS; DFS	Yes; Yes	33.1 (IQR, 25.6–49.68)
Wang (4), 2024 (30)	60 (IQR, 53-65)	145/91	MSR	363.906	I-III	Surgery	CSS; DFS	Yes; Yes	60 (IQR, 60–60)

IQR, interquartile range; SEMS, self-expandable metallic colonic stent; ROC, receiver operating characteristic; MSR, maximally selected rank; OS, overall survival; DFS, disease-free survival; RFS, recurrence-free survival; PFS, progression-free survival; CSS, cancer-specific survival; NR, not reported.

### 3.2 Relationship between the pretreatment PIV and OS

**Figure 2:**
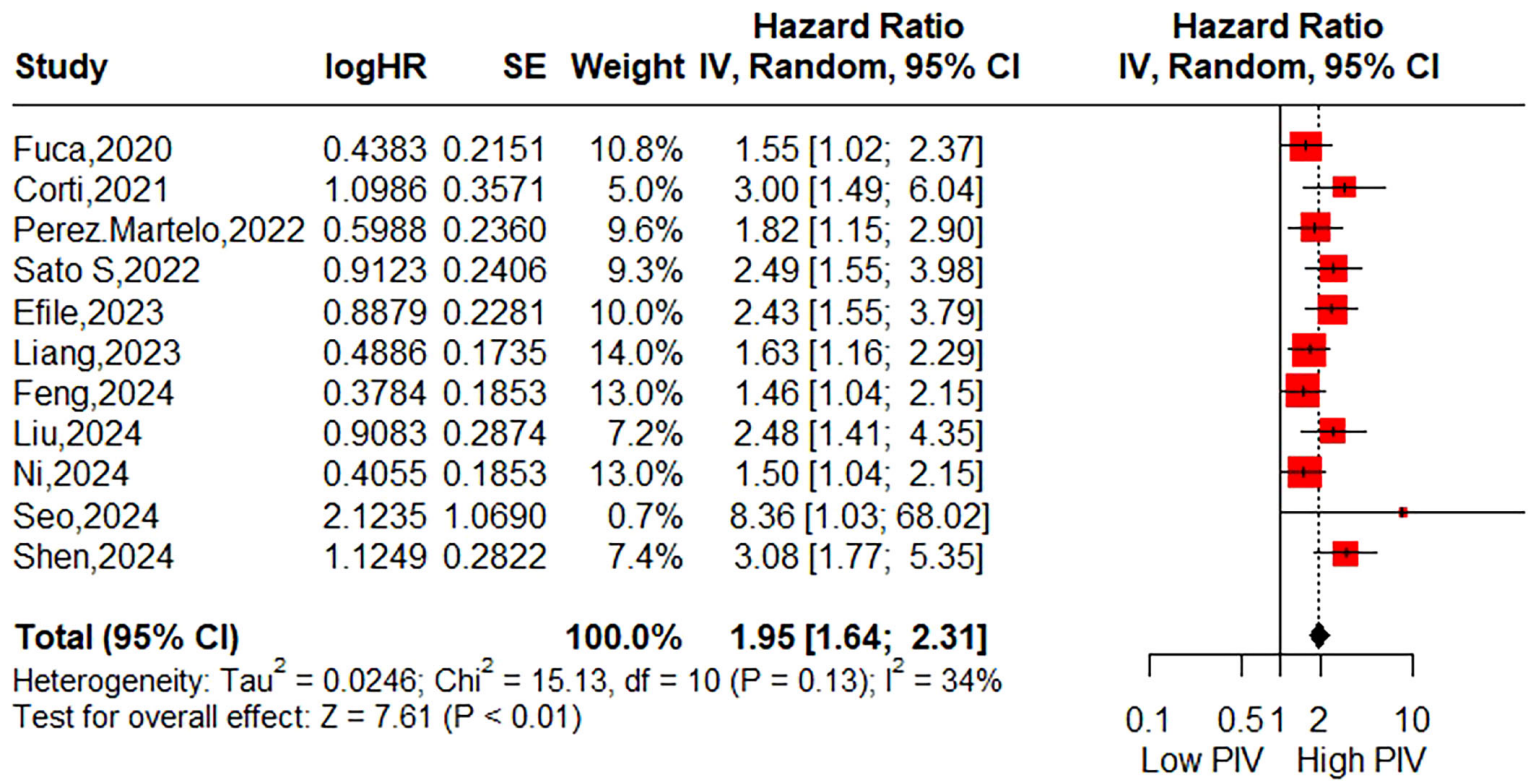
Forest plot assessing the relationship between the pretreatment PIV and overall survival.

The association between the pretreatment PIV and OS was evaluated in 11 studies involving 3,681 patients. The pooled HR was 1.95 (95% CI: 1.64-2.31; P < 0.01), indicating a significant correlation between higher PIV and poorer OS in CRC patients (**Figure 2**). Additionally, subgroup analyses were performed to assess the robustness of the pooled result across various factors, including country (East Asian vs. Others), sample size (<300 vs. >300), sampling time (Within one week vs. Beyond one week vs. Not reported), exclusion of patients with diseases affecting biomarker testing (Yes vs. Not reported), selection method for cut-off value (ROC curve vs. MSR vs. Others), cut-off value (<300 vs. >300), tumor location (Colorectal cancer vs. Colon cancer vs. Rectal cancer), tumor stage (Non-metastatic vs. Mixed vs. Metastatic), treatment strategy (Surgery vs. Neoadjuvant vs. Systemic therapy), and NOS score (6 vs. 7). As shown in **Table 3** and **Supplementary Figure S1**, all subgroup analyses consistently revealed that patients with higher PIV had significantly reduced OS compared to those with lower PIV.

**Table 3:** Subgroup analyses for OS of PIV-high patients vs. PIV-low patients.

Subgroup	Cohorts	Patients	Pooled analysis		I square (%)
			HR	95%CI	
**All patients**	**11**	3681	1.95	1.64-2.31	34
Country					
East Asian	7	2646	1.94	1.52-2.48	47
Others	4	1035	2.00	1.54-2.60	16
Sample size					
<300	6	991	2.20	1.61-3.02	44
>300	5	2690	1.81	1.48-2.21	25
Blood sampling time					
Within one week	5	1664	1.64	1.35-1.99	31
Beyond one week	3	649	2.32	1.76-3.07	5
Not reported	3	1368	2.06	1.48-2.88	26
Excluding patients with diseases affecting biomarker test					
Yes	5	1690	2.01	1.49-2.71	54
Not reported	6	1991	1.89	1.55-2.31	22
Selection method for cut-off value					
ROC curve	4	2056	1.66	1.37-2.20	17
MSR	3	731	1.84	1.38-2.44	20
Others	4	894	2.68	2.00-3.59	0
Cut-off value					
<300	3	1128	2.04	1.31-3.17	44
>300	8	2553	1.95	1.59-2.40	39
Tumor location					
Colorectal cancer	9	3162	1.77	1.52-2.06	21
Colon cancer	1	304	2.43	1.55-3.79	–
Rectal cancer	1	215	3.08	1.77-5.35	–
Tumor stage					
Non-metastatic	3	1277	2.60	1.97-3.45	0
Mixed	5	1673	1.64	1.36-1.99	20
Metastatic	3	731	1.84	1.38-2.44	20
Treatment strategy					
Surgery	7	2735	1.87	1.52-2.30	36
Systematic	3	731	1.84	1.38-2.44	20
Neoadjuvant	1	215	3.08	1.77-5.35	–
NOS					
6	2	601	2.03	1.07-3.83	60
7	9	3080	1.96	1.62-2.37	37

ROC, receiver operating characteristic; MSR, maximally selected rank; NOS, Newcastle-Ottawa Scale; HR, hazard ratio; CI, confidence interval.

Bold values means parameters of subgroup analysis.

### 3.3 Relationship between the pretreatment PIV and DFS

**Figure 3:**
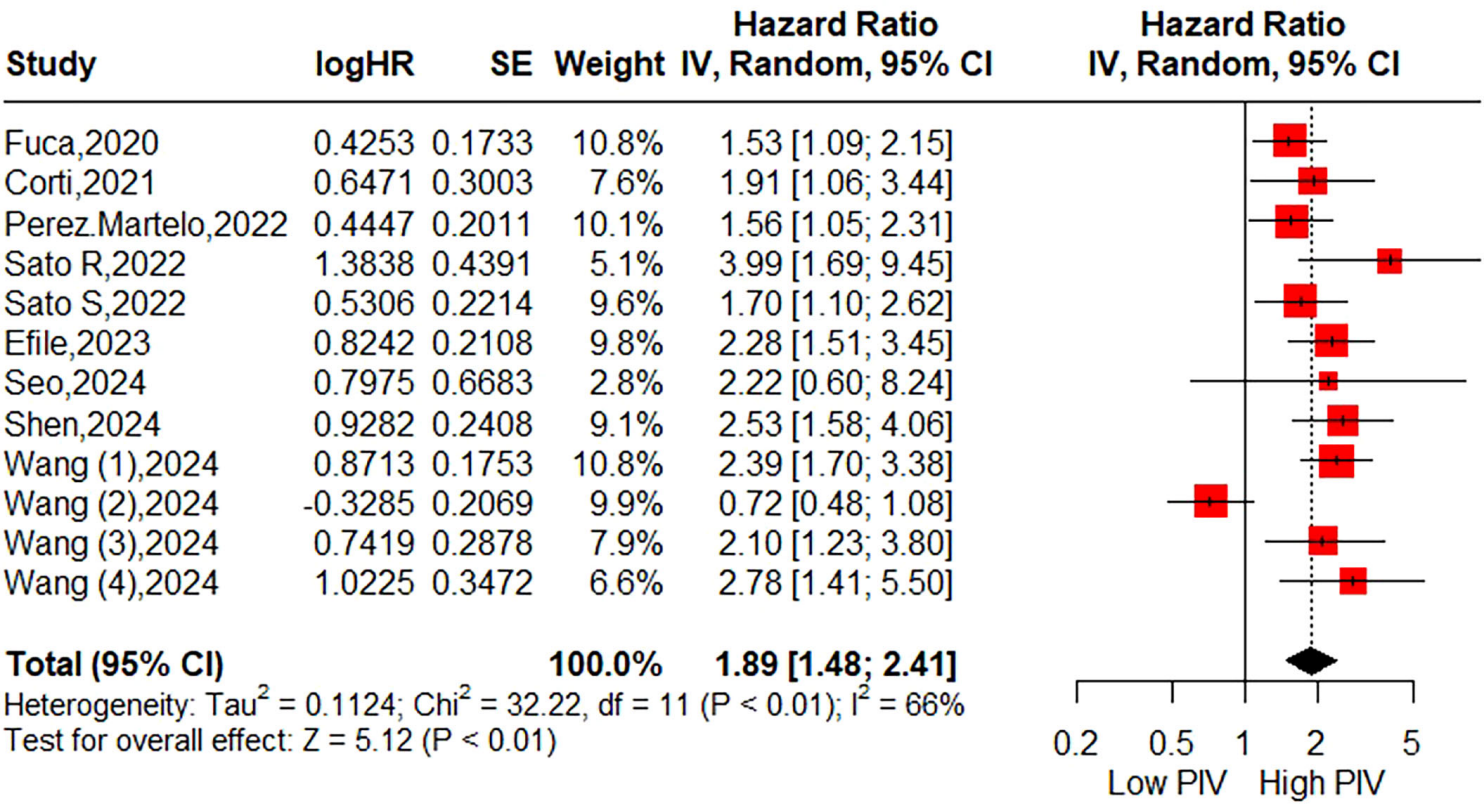
Forest plot accessing the relationship between the pretreatment PIV and disease-free survival.

A total of ten studies (12 cohorts) involving 4,722 patients reported on DFS. The pooled HR was HR=1.89 (95%CI: 1.48-2.41; P<0.01; I^2^ = 66%), indicating a significant association between higher PIV and poorer DFS (**Figure 3**). As shown in **Table 4** and **Supplementary Figure S2**, subgroup analyses based on the aforementioned factors revealed that patients in the higher PIV group experienced worse DFS across most subpopulations, with exceptions including subgroups with a cut-off value < 300 (HR = 1.86; 95% CI: 0.85–4.06), colon cancer (HR = 1.58; 95% CI: 0.74–3.41), and mixed tumor stages (HR = 2.22; 95% CI: 0.60–8.24). Sensitivity analysis demonstrated that the pooled DFS remained statistically significant when omitting each study at a time, and its heterogeneity was substantially reduced (I² = 3%) upon exclusion of the second cohort reported by Wang et al. (30). (**Supplementary Figure S3**).

**Table 4:** Subgroup analyses for DFS of PIV-high patients vs. PIV-low patients.

Subgroup	Cohorts	Patients	Pooled analysis		I square (%)
			HR	95%CI	
**All patients**	**12**	4722	1.89	1.48-2.41	66
Country					
East Asian	8	3687	2.00	1.37-2.92	76
Others	4	1035	1.74	1.42-2.14	0
Sample size					
<300	6	1033	2.19	1.65-2.91	12
>300	6	3689	1.66	1.16-2.38	79
Blood sampling time					
Within one week	4	1281	2.19	1.56-3.07	0
Beyond one week	3	649	2.04	1.52-2.74	30
Not reported	5	2354	1.69	1.01-2.81	84
Excluding patients with diseases affecting biomarker test					
Yes	3	1059	2.32	1.53-3.51	43
Not reported	9	3225	1.76	1.32-2.35	70
Selection method for cut-off value					
ROC curve	2	844	2.39	1.05-5.43	67
MSR	7	3156	1.68	1.20-2.35	75
Others	3	722	2.38	1.76-3.22	0
Cut-off value					
<300	4	1799	1.86	0.85-4.06	88
>300	8	2923	1.89	1.69-2.22	0
Tumor location					
Colorectal cancer	6	1778	1.71	1.40-2.09	0
Colon cancer	3	1814	1.58	0.74-3.41	91
Rectal cancer	3	1130	2.43	1.77-3.35	0
Tumor stage					
Non-metastatic	8	3778	2.02	1.42-2.86	77
Mixed	1	203	2.22	0.60-8.24	–
Metastatic	3	293	1.60	1.26-2.02	0
Treatment strategy					
Surgery	8	3776	1.97	1.36-2.85	76
Systematic	3	293	1.60	1.26-2.02	0
Neoadjuvant	1	731	2.53	1.58-4.06	–
NOS					
6	3	687	1.99	1.25-3.19	52
7	9	4035	1.85	1.38-2.49	71

ROC, receiver operating characteristic; MSR, maximally selected rank; NOS, Newcastle-Ottawa Scale; HR, hazard ratio; CI, confidence interval.

Bold values means parameters of subgroup analysis.

### 3.4 Relationship between the pretreatment PIV and CSS

**Figure 4:**
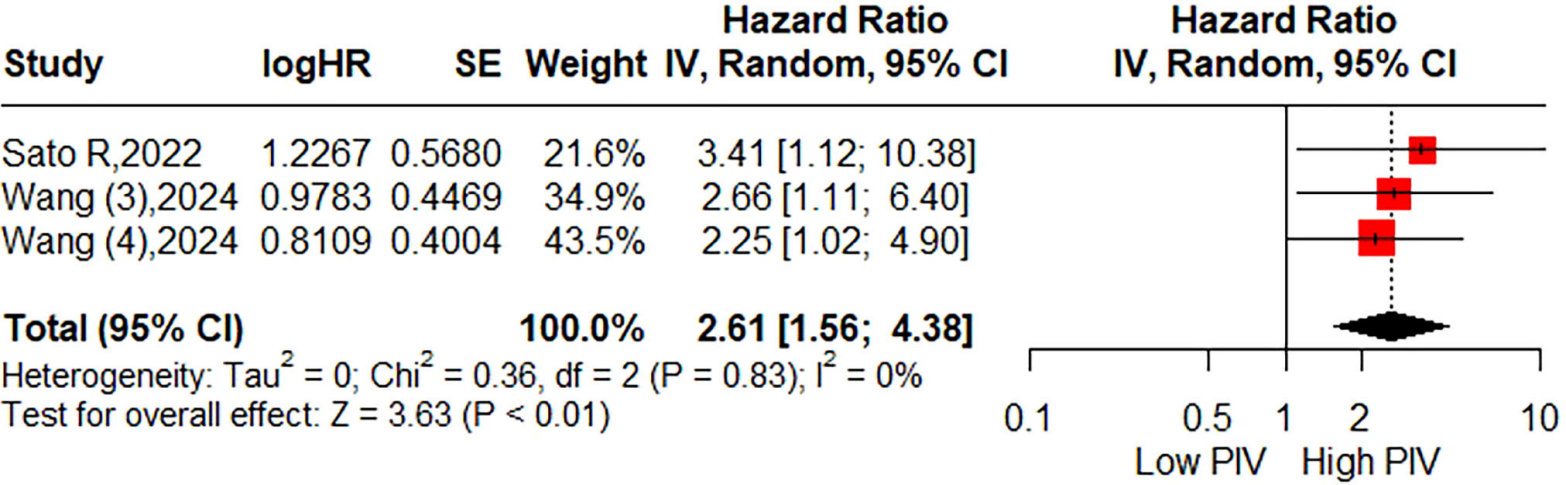
Forest plot accessing the relationship between the pretreatment PIV and cancer-specific survival.

The relationship between the pretreatment PIV and CSS was assessed in two studies (3 cohorts) involving 1,001 patients. The pooled HR was 2.61 (95% CI: 1.56-4.38; P < 0.01; I^2^ = 0%), suggesting a possible association between higher PIV and relatively poorer CSS (**Figure 4**). Due to the limited number of included studies, subgroup analyses were not conducted.

### 3.5 Publication bias

**Figure 5:**
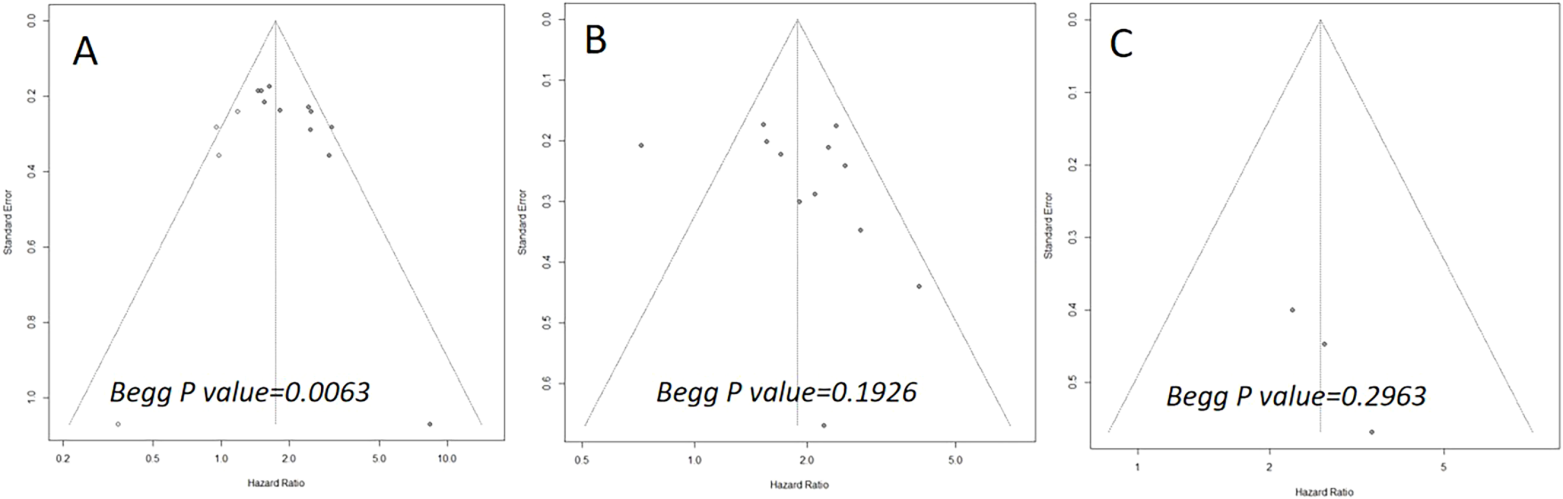
Begg’s funnel plots assessing publication bias between the pretreatment PIV and overall survival **(A)**, disease-free survival **(B)**, and cancer-specific survival **(C)**.

The Begg’s funnel plots are illustrated in **Figure 5**. The results of the Begg’s test indicated a significant publication bias concerning OS (P=0.0063). However, the trim-and-fill analysis revealed that the pooled result remained robust after accounting for four additional hypothetical unpublished studies (HR=1.74; 95% CI: 1.45-2.09; P<0.01; I^2^ = 50.8%). For DFS and CSS, the funnel plots exhibited bilateral symmetry, with P values of 0.1926, and 0.2963, respectively, as determined by the Begg’s test.

## 4 Discussion

Cancer-related inflammation is pervasive among patients with malignant diseases and has been firmly established as a driving force in cancer progression and metastasis (31). Traditionally, the host’s inflammatory state could be reflected by blood-based biomarkers to some extent, including neutrophil count, platelet count, and lymphocyte count (32). Moreover, extensive evidence from numerous studies underscores the predictive power of their ratios for evaluating both short-term and long-term patient outcomes, particularly within the context of oncology (8). Notably, these markers possess intrinsic advantages—being non-invasive, objective, and economically viable—thereby offering great promise for broad clinical implementation (2).

In recent years, a novel biomarker known as the PIV—a composite indicator encompassing serum neutrophils, platelets, monocytes, and lymphocytes—has garnered significant attention from clinicians (11). This is largely due to its remarkable prognostic potential reported in various malignancies. In ovarian cancer, Liao et al. (33) demonstrated that patients in the high PIV group exhibited poorer OS and PFS compared to those in the low PIV group. In breast cancer, Li et al. (34) revealed that the pretreatment PIV was a useful predictive indicator for pathological complete response and long-term survival in patients undergoing neoadjuvant chemotherapy. Furthermore, a recent meta-analysis by Kuang et al. (35) confirmed that elevated PIV levels are associated with reduced OS and PFS in cancer patients receiving immune checkpoint inhibitors. In CRC, although a previous meta-analysis by Yang et al. (15) in 2022 showed the significant efficacy of PIV in predicting long-term survival, this study incorporated only 6 studies with 1,879 patients, which may limit the clarity and generalizability of its conclusions. Therefore, further investigation into the prognostic value of PIV in CRC patients remains essential.

By synthesizing data from 14 studies encompassing a total of 6,192 CRC patients, our meta-analysis demonstrated that patients in the high PIV group exhibited a 1.95-fold increased risk of poor OS. Furthermore, subgroup analyses conducted based on eligible factors reinforced the prognostic significance of PIV across patients with diverse clinical characteristics. Although significant publication bias was detected, the trim-and-fill method consistently corroborated the robustness of the pooled results. Meanwhile, this meta-analysis revealed that patients in the high PIV group faced a 1.89-fold increased risk of poor DFS, with no substantial publication bias observed. Subgroup analyses indicated that pretreatment PIV held significant prognostic value in most subsets, except for subgroups defined by a cut-off value < 300, colon cancer, and mixed tumor stages. These inconsistent findings in certain subgroups should be interpreted with caution due to the limited number of cohorts included in these analyses. Sensitivity analysis further identified that these inconsistencies primarily stemmed from the second cohort reported by Wang et al. (30), which suggested limited prognostic utility of PIV for DFS in patients with right-sided colon cancer (HR = 0.72; 95% CI: 0.48–1.06). Upon excluding this cohort, the heterogeneity (I²) markedly decreased from 66% to 3%. Additionally, two studies initially explored the association between PIV and CSS, yielding statistically significant outcomes. Compared to prior meta-analysis, the primary strength of the current study lies in its inclusion of a more heterogeneous population with varied clinical features, thereby enhancing the generalizability of PIV’s prognostic value.

The potential mechanism by which the PIV can effectively predict prognosis in CRC patients can be explained through the following aspects. First, neutrophils, which are the most common innate immune cells, have been documented to facilitate tumor invasion and metastasis through the secretion of vascular endothelial growth factor A (VEGFA), matrix metalloproteinases (MMPs), and other chemokines such as interleukin-6 (IL-6) and transforming growth factor-beta (TGF-β) (36). Additionally, elevated levels of neutrophils can impair T cell activation by releasing substantial amounts of nitric oxide, arginase, and reactive oxygen species, thereby suppressing the body’s cytotoxic effect on cancer cells (37). Second, monocytes, particularly those that differentiate into tumor-associated macrophages (TAMs), can induce apoptosis in antitumor T cells (38). Furthermore, TAM density has been shown to influence tumor tissue angiogenesis by promoting the production and secretion of pro-angiogenic factors (39). Third, platelets have been reported to induce epithelial-mesenchymal transition (EMT) and angiogenesis via the secretion of TGF-β, VEGF, and fibroblast growth factor (FGF) (40). Moreover, platelets can recruit neutrophils and monocytes, thus facilitating the distant metastasis of tumor cells (32). Finally, lymphocytes serve as the primary effector cells of the immune system, coordinating immune responses against tumor cells (41). Tumor-infiltrating lymphocytes secrete a variety of cytokines, such as IFN-γ and TNF-α, which not only inhibit tumor growth but also promote tumor cell apoptosis (42). Specifically, CD8+ T cells can directly induce tumor cell death through the release of perforin and granzyme (43). Consequently, a reduction in lymphocyte count impairs the body’s capacity to effectively suppress tumor progression.

The present meta-analysis is subject to several notable limitations that warrant careful consideration. Firstly, all studies incorporated into this analysis were retrospective in nature, inherently carrying the risk of selection bias. This underscores the critical need for future prospective studies to establish a causal direction. Secondly, the majority of the studies were from East Asia, introducing a potential regional bias and highlighting the imperative for greater global diversity in future research endeavors. Thirdly, the study by Wang et al. (30) demonstrated that the pretreatment PIV does not exhibit prognostic value for DFS in right-sided colon cancer. And similar findings were observed in their study for other inflammatory markers, such as the NLR, PLR and systemic immune-inflammation index. However, the underlying biological mechanisms remain unclear. We contend that further clinical and basic research is warranted to elucidate the role of PIV in right-sided colon cancer. Lastly, although we preliminarily investigated the significant relationship between the pre-treatment PIV and CSS in colorectal cancer patients, our finding was derived solely from a meta-analysis of two studies. Consequently, additional studies are warranted to validate this issue.

## 5 Conclusions

Our research reveals that the pretreatment PIV could function as a remarkably insightful prognostic biomarker for individuals diagnosed with colorectal cancer, as individuals in the high PIV group demonstrate significantly poorer long-term survival outcomes. By harnessing this promising indicator, clinicians may effectively categorize patients and design more precise, individualized therapeutic approaches. However, further rigorous investigation is indispensable to substantiate the reliability and applicability of this biomarker in colorectal cancer prognosis.

## Data Availability

The original contributions presented in the study are included in the article/**Supplementary Material**. Further inquiries can be directed to the corresponding authors.
