# Supplementary material for: Prognostic significance of the pretreatment pan-immune-inflammation value in colorectal cancer patients: an updated meta-analysis
Source: Front Oncol. 2025 Jul 24;15:1599075. doi: 10.3389/fonc.2025.1599075 (PMC12328146; doi:10.3389/fonc.2025.1599075)
Supplement: Supplementary file 1 [file DataSheet1.docx]

Table S1. Quality assessment of included studies.

| **Reference** | **Representatives of the exposed cohort** | **Selection of the non-exposed cohort** | **Ascertainment of exposure** | **Was outcome of interest present at start of study** | **Comparability of cohorts on the basis of the design or analysis** | **Assessment of outcome** | **Was follow-up long enough for outcomes to occur** | **Adequate follow up** | **Total** |
| --- | --- | --- | --- | --- | --- | --- | --- | --- | --- |
| Fuca, 2020 | 1 | 1 | 1 | 0 | 1 | 1 | 0 | 1 | 6 |
| Corti, 2021 | 1 | 1 | 1 | 0 | 1 | 1 | 0 | 1 | 6 |
| Perez‑Martelo, 2022 | 1 | 1 | 1 | 0 | 1 | 1 | 1 | 1 | 7 |
| Sato R, 2022 | 1 | 1 | 1 | 0 | 1 | 1 | 0 | 1 | 6 |
| Sato S, 2022 | 1 | 1 | 1 | 0 | 1 | 1 | 1 | 1 | 7 |
| Efile, 2023 | 1 | 1 | 1 | 0 | 1 | 1 | 1 | 1 | 7 |
| Liang, 2023 | 1 | 1 | 1 | 0 | 1 | 1 | 1 | 1 | 7 |
| Feng, 2024 | 1 | 1 | 1 | 0 | 1 | 1 | 1 | 1 | 7 |
| Liu, 2024 | 1 | 1 | 1 | 0 | 1 | 1 | 1 | 1 | 7 |
| Ni, 2024 | 1 | 1 | 1 | 0 | 1 | 1 | 1 | 1 | 7 |
| Seo, 2024 | 1 | 1 | 1 | 0 | 1 | 1 | 1 | 1 | 7 |
| Shen, 2024 | 1 | 1 | 1 | 0 | 1 | 1 | 1 | 1 | 7 |
| Wang (1), 2024 | 1 | 1 | 1 | 0 | 1 | 1 | 1 | 1 | 7 |
| Wang (2), 2024 | 1 | 1 | 1 | 0 | 1 | 1 | 1 | 1 | 7 |
| Wang (3), 2024 | 1 | 1 | 1 | 0 | 1 | 1 | 1 | 1 | 7 |
| Wang (4), 2024 | 1 | 1 | 1 | 0 | 1 | 1 | 1 | 1 | 7 |


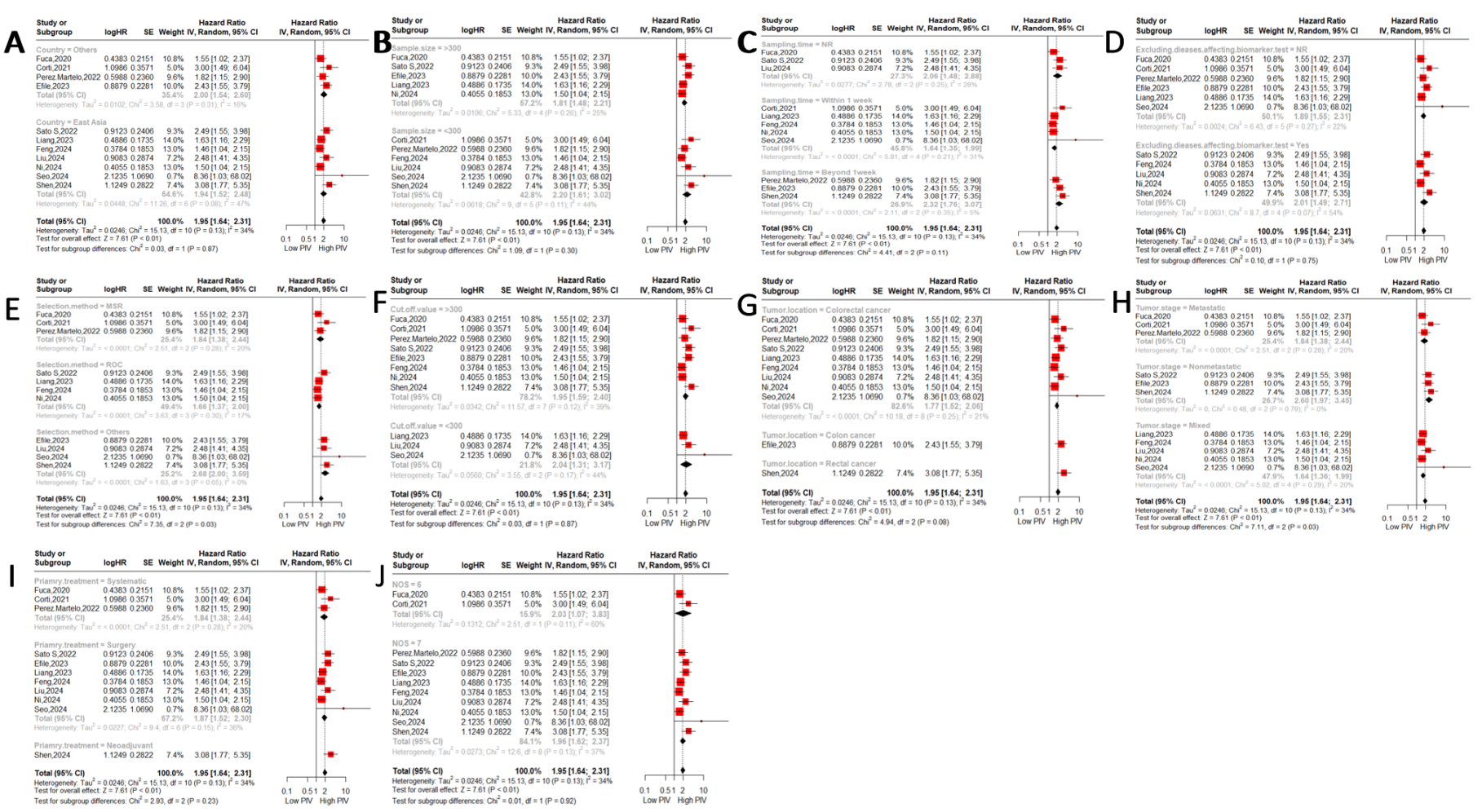


Figure S1. Forest plot of subgroup analyses assessing the relationship between the PIV and overall survival. A: Country (East Asia vs. Others); B: Sample size (>300 vs. <300); C: Blood sampling time (Within one week vs. Beyond one week vs. Not reported); D: Excluding patients with diseases affecting biomarker test (Yes vs. Not reported); E: Selection method for cut-off value (ROC curve vs. MSR vs. Others); F: Cut-off value (>300 vs. <300); G: Tumor location (Colorectal cancer vs. Colon cancer vs. Rectal cancer); H: TNM stage (Non-metastatic vs. Mixed vs. Metastatic); I: Primary treatment (Surgery vs. Systematic vs. Neoadjuvant); J: NOS (6 vs. 7).


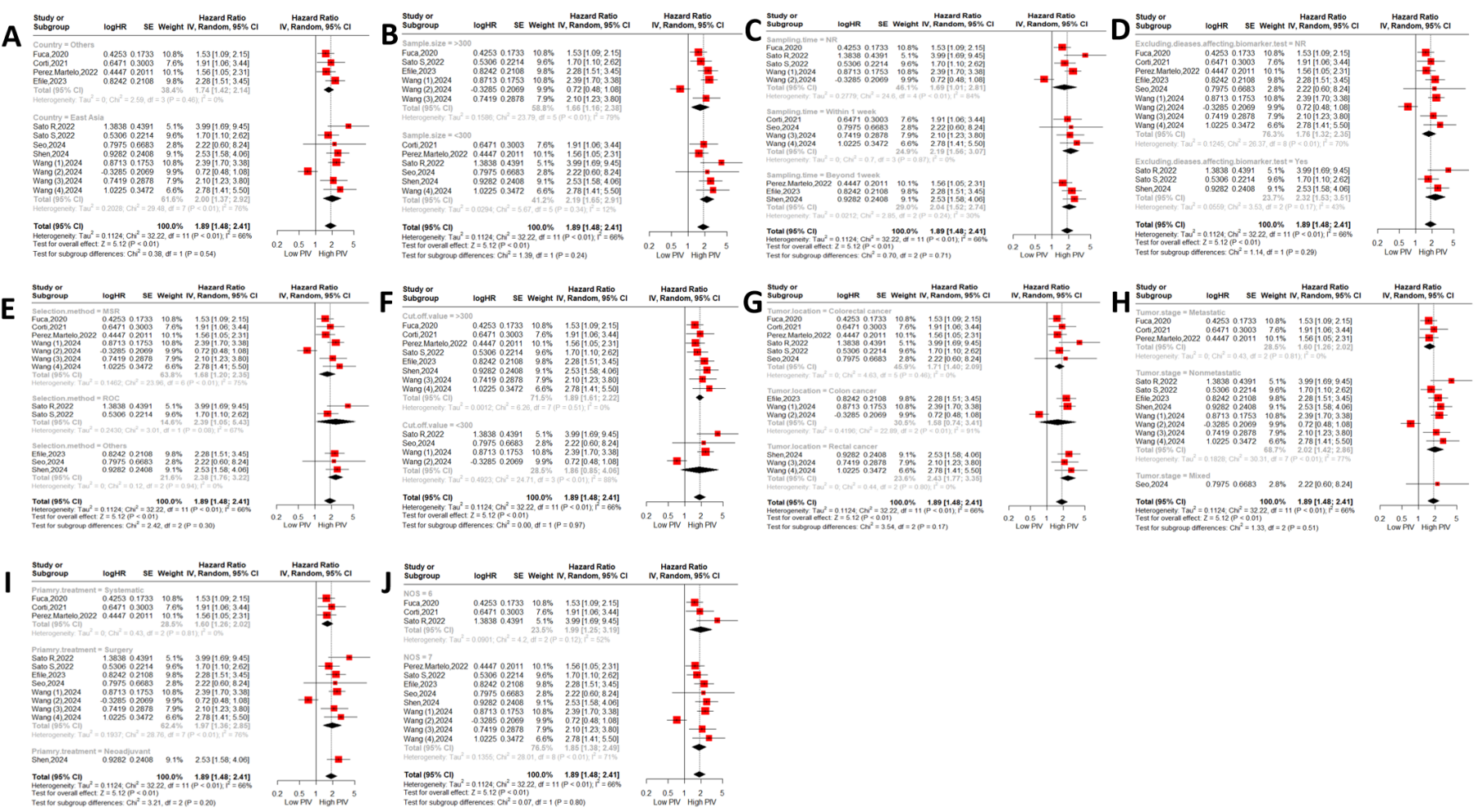


Figure S2. Forest plot of subgroup analyses assessing the relationship between the PIV and overall survival. A: Country (East Asia vs. Others); B: Sample size (>300 vs. <300); C: Blood sampling time (Within one week vs. Beyond one week vs. Not reported); D: Excluding patients with diseases affecting biomarker test (Yes vs. Not reported); E: Selection method for cut-off value (ROC curve vs. MSR vs. Others); F: Cut-off value (>300 vs. <300); G: Tumor location (Colorectal cancer vs. Colon cancer vs. Rectal cancer); H: TNM stage (Non-metastatic vs. Mixed vs. Metastatic); I: Primary treatment (Surgery vs. Systematic vs. Neoadjuvant); J: NOS (6 vs. 7).


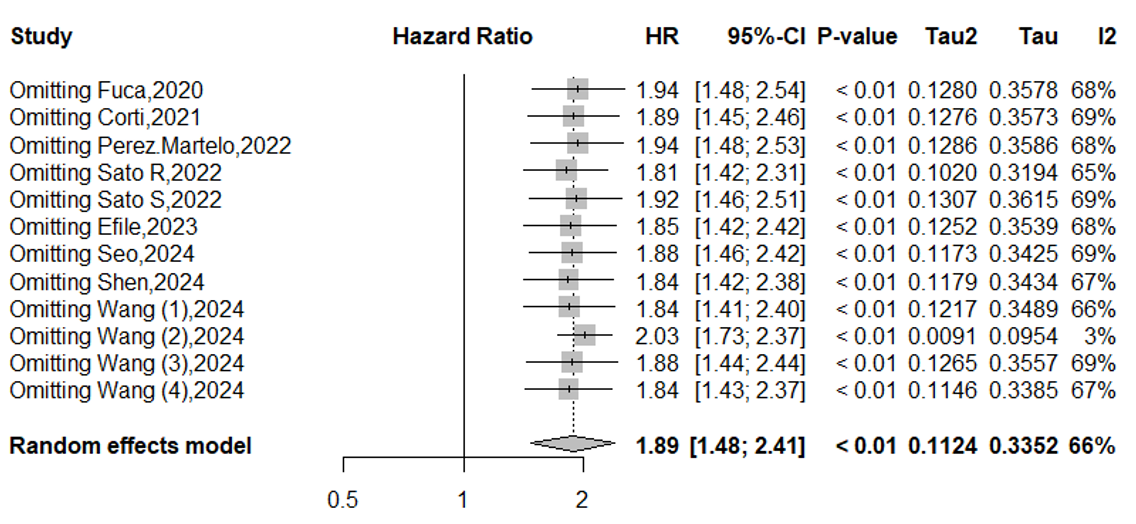


Figure S3. Sensitivity analysis assessing disease-free survival.
